# Combined Targeting of Pathogenetic Mechanisms in Pancreatic Neuroendocrine Tumors Elicits Synergistic Antitumor Effects

**DOI:** 10.3390/cancers14225481

**Published:** 2022-11-08

**Authors:** Sebastian Gulde, Alessia Foscarini, Simon L. April-Monn, Edoardo Genio, Alessandro Marangelo, Swapna Satam, Daniel Helbling, Massimo Falconi, Rodrigo A. Toledo, Jörg Schrader, Aurel Perren, Ilaria Marinoni, Natalia S. Pellegata

**Affiliations:** 1 Institute for Diabetes and Cancer, Helmholtz Zentrum München, Ingolstaedter Landstrasse 1, 85764 Neuherberg, Germany; 2 Joint Heidelberg-IDC Translational Diabetes Program, Heidelberg University Hospital, 69120 Heidelberg, Germany; 3 Department of Biology and Biotechnology “L. Spallanzani”, University of Pavia, 27100 Pavia, Italy; 4 Institute of Pathology, University of Bern, 3012 Bern, Switzerland; 5 OnkoZentrum Zurich, 8038 Zurich, Switzerland; 6 Pancreatic Surgery Unit, Pancreas Translational and Clinical Research Centre, IRCCS San Raffaele Scientific Institute, 20132 Milan, Italy; 7 CIBERONC, Gastrointestinal and Endocrine Tumors, VHIO, 08035 Barcelona, Spain; 8 Department of Medicine, University Medical Center Hamburg-Eppendorf, 20251 Hamburg, Germany; 9 Department of Medicine, Klinikum Nordfriesland, 25813 Husum, Germany

**Keywords:** pancreatic NETs, buparlisib, ribociclib, combination therapy, primary human tumoroids

## Abstract

**Simple Summary:**

Pancreatic neuroendocrine tumors (PanNETs) are often diagnosed when advanced or metastatic, and at this stage curative surgery in no longer an option. Given that available treatments for advanced disease have shown limited efficacy, novel therapies are urgently needed. In this scenario, we selected two drugs, inhibiting pathways known to be activated in PanNETs, and evaluated their efficacy in various preclinical tumor models. We chose a PI3K inhibitor (buparlisib) and a CDK4/6 inhibitor (ribociclib). We first tested these drugs, alone or in combination, on established cell lines representing distinct PanNET differentiation states. The combination buparlisib plus ribociclib reduced the proliferation of the cell lines more effectively than the single drugs. Inhibition of downstream target genes and/or proteins explained the drugs’ anti-proliferative activity. Buparlisib, but not ribociclib, promoted cell death. We then demonstrated that the combination treatment with buparlisib and ribociclib inhibits the viability of primary islets from a genetic animal model of PanNETs (*Men1*-deficient mice), without significantly affecting viability and function of primary islets from wild-type mice. Noteworthy, treatment of primary patient-derived PanNET cultures supported the efficacy of the combination treatment. Our findings indicate that the combined inhibition of PI3K and CDK4/6 pathways is a potentially effective therapeutic option for PanNETs.

**Abstract:**

Pancreatic neuroendocrine neoplasms (PanNENs) are the second most common malignancy of the pancreas. Surgery remains the only curative treatment for localized disease. For patients with inoperable advanced or metastatic disease, few targeted therapies are available, but their efficacy is unpredictable and variable. Exploiting prior knowledge on pathogenetic processes involved in PanNEN tumorigenesis, we tested buparlisib (PI3K inhibitor) and ribociclib (CDK4/6 inhibitor), as single agents or in combination, in different preclinical models. First, we used cell lines representative of well-differentiated (INS-1E, NT-3) and poorly differentiated (BON-1) PanNENs. The combination of buparlisib with ribociclib reduced the proliferation of 2D and 3D spheroid cultures more potently than the individual drugs. Buparlisib, but not ribociclib, induced apoptosis. The anti-proliferative activity of the drugs correlated with downstream target inhibition at mRNA and protein levels. We then tested the drugs on primary islet microtissues from a genetic PanNET animal model (*Men1*-defective mice) and from wild-type mice: the drug combination was effective against the former without altering islet cell physiology. Finally, we treated PanNET patient-derived islet-like 3D tumoroids: the combination of buparlisib with ribociclib was effective in three out of four samples. Combined targeting of PI3K and CDK4/6 is a promising strategy for PanNENs spanning various molecular and histo-pathological features.

## 1. Introduction

Pancreatic neuroendocrine neoplasms (PanNENs) account for <3% of all pancreatic tumors, but their incidence has been increasing in recent decades [1], in part due to more accurate diagnosis. PanNENs are classified as functioning or non-functioning depending on whether they cause symptoms of hormonal hypersecretion, with the latter group accounting for 60–90% of cases [2]. Among functioning PanNET, insulinoma are most frequent. PanNENs are characterized by a heterogeneous and unpredictable clinical behavior, which depends on their stage of progression, pathological grade and hormone secretion. PanNETs are usually indolent tumors, but they all have malignant potential. To better stratify patients for prognostic purposes, the World Health Organization (WHO) introduced a new classification of PanNENs in 2017, which divides these neoplasms into grade 1 (G1) to G3 pancreatic neuroendocrine tumors (PanNETs), and G3 neuroendocrine carcinomas (PanNECs). The classification in the various grades is based on proliferation rate (i.e., mitotic count and Ki67 index), histomorphology and molecular biomarkers [2]. The grading of the tumors has a significant impact on the overall survival of the patients, which ranges from >10 years for G1 PanNETs, to approximately 6 years for G2 tumors, to less than 10 months for aggressive PanNECs [3,4].

Surgical resection is the first-line and the only potentially curative treatment for patients with localized disease. Medical treatment for patients with unresectable or metastatic PanNETs includes somatostatin analogues (SSAs) as first-line therapy, and either everolimus (mTOR inhibitor), sunitinib (multikinase inhibitor), temozolomide, streptozocin, or peptide receptor radionuclide therapy (PRRT) with radiolabeled SSAs as second-line treatments [5]. However, none of these treatment options are curative, and only a fraction of patients treated profit. Therefore, the identification of more effective, targeted therapies for aggressive PanNENs is highly clinically relevant.

Although mostly sporadic, PanNET can develop as a component of hereditary multi-tumor syndromes, including multiple endocrine neoplasia type 1 (MEN1), von Hippel-Lindau (VHL) disease, and, more rarely, neurofibromatosis type 1 (NF-1), and tuberous sclerosis (TSC) [6]. MEN1 syndrome is caused by inactivating germline mutations of the MEN1 tumor suppressor gene [7]. MEN1 is also mutated in 40% of sporadic, well-differentiated PanNETs [6]. The importance of the *Men1* gene as the driver of PanNET tumorigenesis has been demonstrated by studies of mice with defective *Men1* function (heterozygous knockout *Men1*^+/−^ mice), where PanNET development was observed at high incidence during their life-span, thereby recapitulating the situation seen in MEN1 patients [8]. Recent NGS studies have shed light into the somatic mutations playing a role in PanNET development and progression [9,10]. Inactivation of *ATRX/DAXX* and hyperactivation of the PI3K/AKT/mTOR pathway are recurring features in PanNET tumorigenesis. The latter is driven by mutations in mTOR-related genes (e.g., *PI3CA*, *TSC2*, *PTEN*, *DEPDC5)*, by the loss of chromosomal regions containing *TSC2* (16p) and *PTEN* (10q23), by overexpression of various tyrosine kinase receptors, and by the activation of Akt [11,12]. PI3K/AKT/mTOR pathway activation correlates with worse patient outcome [10].

Inactivation of the retinoblastoma pathway was originally implicated in PanNET tumorigenesis based on studies of double knockout mice having inactivation of Rb1 and Tp53: Tp53^+/−;^ Rb^+/−^ and Tp53^−/−^; Rb^+/−^ mice developed non-invasive islet carcinoma, together with other neuroendocrine and non-neuroendocrine tumors [13]. The cyclin-dependent kinases CDK4 and CDK6 phosphorylate Rb1 and inhibit its function. Gene amplification and overexpression of CDK4 and CDK6 was demonstrated in the majority of PanNET patients [14]. Interestingly, the deletion of *Cdk4* in *Men1*^+/−^ mice (*Men1*^+/−^; *Cdk4*^−/−^ mice) abrogated PanNET formation, thereby suggesting that CDK4 is a critical downstream target of Men1-dependent tumorigenesis [15]. Repression by promoter methylation of p16INK4a, a cyclin-dependent kinase (CDK) inhibitor that enforces RB1 tumor-suppressive activity by inhibiting its phosphorylation by CDK4 and CDK6, is common in PanNETs [12]. These data support a role for CDKs and cell cycle regulation in PanNENs.

Given the relevance of an overactivation of the PI3K pathway in several cancers, agents that can block this signaling cascade at various levels have been generated and several are already in clinics. Buparlisib, a PI3K inhibitor, has been evaluated for its anti-tumor efficacy in human and rodent PanNET cell lines in vitro, and was found to inhibit cell proliferation and induce apoptosis as a single agent [16,17,18]. Buparlisib in combination with streptozotocin also showed antitumor effects in vivo in a xenograft model of liver dissemination obtained upon intrasplenic INS-1E cells injection [16].

Among the available CDK4/6 inhibitors, palbociclib as a monotherapy was evaluated in a small number of unselected and heavily pretreated patients with G1/2 PanNETs [19]. This trial failed to show the therapeutic effects of this drug. However, it brought to light the need for a molecular-based patient stratification: given the heterogeneity of PanNETs, a stratification based on the genetic mutations (e.g., in *MEN1*) is needed to select the patients that might benefit from this treatment. Therefore, further evaluation of CDK4/6 inhibitors against PanNETs in the clinics is warranted [20].

The aim of our study was to identify a novel and effective treatment strategy for PanNENs by exploiting the knowledge of relevant pathogenetic mechanisms involved in these tumors. By using different preclinical in vitro models, including patient-derived primary 3D tumoroids, we report that the combination treatment of a small-molecule, orally available, pan-class I PI3K inhibitor (buparlisib) with a CDK4/6 inhibitor (ribociclib) suppresses PanNEN cell growth and holds promise for future clinical implementation.

## 2. Materials and Methods

### 2.1. Cell Lines

INS-1E cells were obtained from Pierre Maechler and maintained in RPMI 1640 Medium GlutaMAX™ (61870044, Life Technologies—Carlsbad, CA, USA) supplemented with 5% FBS (10500064, Life Technologies), 1% Penicillin/Streptomycin (15070063, Life Technologies), 1 mM Pyruvate (11360-039, Life Technologies), 10 mM HEPES (15630-056, Life Technologies), and 50 µM 2-Mercaptoethanol (31350-010, Life Technologies). NT-3 cells were cultivated in RPMI 1640 Medium GlutaMAX™ with 10% FBS, 1% Penicillin/Streptomycin, 20 ng/mL EGF (AF-100-15, Peprotech – Cranbury, NJ, USA), 10 ng/mL FGF (100-18B, Peprotech) on plates coated with 50 µg/mL H_2_O Collagen from human placenta (C7521, Sigma-Aldrich—St. Louis, MI, USA), as previously reported [21]. NT-3 cells carry a homozygous missense mutation of *MEN1* (chromosome 11, position 64572018; c.1621A>G; p.T541A) [21]. The BON1 cells were provided by E.J. Speel, Maastricht, Netherlands and cultured in DMEM/Ham’s F12 (11320033, Life Technologies) with 10% FBS and 1% Penicillin/Streptomycin.

### 2.2. Primary Human Cultures

All subjects involved in the study gave consent and primary cell cultures have been approved by the cantonal ethic commission Bern, projects ID 105-2015 and ID 2019-01154. Patient samples were isolated and cultured following the described protocol [22]. Cryopreserved tumor tissues of four PanNET patients were used for in vitro drug screening. In short, washed pieces of 1 mm^3^ were dissociated in digestion medium (10 mg/mL collagenase IV (Worthington, Columbus, OH, USA), 0.25% Trypsin-EDTA (Sigma-Aldrich), 0.2 mg/mL DNase (Roche—Basel, Switzerland) in advanced DMEM-F12, Hepes 10 mM, 1% L-glutamine, 1% penicillin-streptomycin-amphotericin B) in a gentle MACS™ dissociator (Miltenyi Biotec, Solothurn, Switzerland). Debris of collagen were removed using a 70-µm strainer, followed by a red blood cell lysis with ACK lysis buffer (Thermo Fisher Scientific, Waltham, MS, USA). Fibroblasts were partially removed, exploiting their differential adhesion capacity to plastic surfaces. Cells were then dissociated into single cells and resuspended and maintained in Advanced DMEM-F12 + GF medium (DMEM-F12, 5% FBS, Hepes 10 mM, 1% L-glutamine (200 mM), 1% penicillin (100 IU/mL), 1% streptomycin (0.1 mg/mL), 1% amphotericin B (0.25 mg/mL) (Merck, Darmstadt, Germany), 20 ng/mL EGF, 10 ng/mL bFGF (Thermo Fisher Scientifi), 100 ng/mL PlGF, and 769 ng/mL IGF-1 (Selleckchem, Boston, MS, USA)), and in 24-well Corning^®^ Costar^®^ ultra-low attachment (ULA) plates (Corning—New York, USA) (500 µL/well, 3–5 × 10^5^ cells/well) in a humidified cell incubator (21% O_2_, 5% CO_2_, 37 °C). After 2 days of recovery phase, cells were counted and resuspended in fresh AdvDMEM + GF medium supplemented with growth-factor-reduced Matrigel and plated in 96-well ULA plates (3–4 × 10^3^ cells/well). For drug screening, isolated cells were resuspended in fresh Advanced DMEM-F12 + GF medium supplemented with 123 µg/mL growth-factor-reduced Matrigel^®^ (Corning) and plated in 96-well ULA plates (50 µL/well, 3–4 × 10^3^ cells/well). RealTime-Glo™ MT Cell Viability (RTG) assay (Promega, Madison, WI, USA) was used to continually monitor cell viability of 3-D human primary PanNET cultures. The RTG assay was performed according to the manufacturer’s instructions, and luminescence was measured in an Infinite^®^ 200 PRO plate reader (Tecan, Männedorf, Switzerland).

### 2.3. Animal Husbandry and Primary Islet-Cell Isolation

Heterozygous knockout mice of the *Men1*^tm12qw^ strain [23] (synonym Men1^T/+^) were bred and maintained in agreement with general husbandry rules approved by the Helmholtz Zentrum München and as approved by the government of Upper Bavaria, Germany (Az 55.2-1-54-2532-117-2016). Mice were killed by cervical dislocation. Islets were isolated by injection of 3 mL CollagenaseP (#11213857001, Roche) solution into the bile duct. Pancreas tissue was digested at 37 °C for 15 min. The reaction was stopped by adding 10 mL ice-cold Hanks’ buffer containing 0.2% bovine serum albumin (#11926.04, Serva-Heidelberg, Germany), followed by filtration using a 500 µm strainer. Islets were hand-picked and single cells were obtained by trypsin incubation. A total of 5000 single cells were then seeded into each well of a hanging drop system (#IPS-06-010, Gravity Plates from Insphero-Schlieren, Switzerland) to obtain 3D microtissues (spheroids) of equal size.

### 2.4. Immunofluorescence of Islets Microtissues (Pseudoislets)

Pseudoislets were transferred to 1.5 mL tubes, centrifuged at 500× *g* for 5 min, and the supernatant was then removed. Islets were fixed for 1 h at RT using 4% Formaldehyde (P087.1, Carl Roth—Karlsruhe, Germany). After washing with PBS, 40 µL of 60 °C HistoGel™ (HG-4000-012, Thermo Fisher) were added to the tubes. The HistoGel-pseudoislet mixture was immediately transferred onto parafilm, allowing it to form a droplet. After it solidified at 4 °C, the droplet was placed in a tissue processing cassette. Dehydration with a standard dehydration program was performed on a tissue processor and the droplet was embedded in paraffin. Immunofluorescence was performed on formalin-fixed paraffin-embedded (FFPE) 4-μm sections as previously described [24]. In brief, sections were deparaffinized, boiled in citric acid, permeabilized and blocked. Then, the sections were incubated with primary antibodies (Supplementary Table S2) overnight at 4 °C and secondary antibodies (Supplementary Table S2) for 1 h at RT. Finally, nuclei were counterstained with DAPI (dilution 1:2000) and the sections were mounted. Images were taken using a confocal microscope (Olympus FluoView 1200; Olympus Corporation).

### 2.5. Drug Treatments and In Vitro Assays

Buparlisib (HY-70063, MedChemExpress—Monmouth Junction, NJ, USA) and ribociclib (HY-15777, MedChemExpress) were dissolved in DMSO and used at the concentrations indicated in the figures. The 2D proliferation was measured after 72 h of treatment with drugs or DMSO controls using the CyQUANT^®^ NF kit (#C35006, Thermo Fisher Scientific) and following the manufacturer’s instructions.

Apoptosis was measured by assessing the Caspase 9 activity in treated cells after 72 h using the Caspase-Glo^®^ 9 Assay System (#G8211, Promega) following the manufacturer’s instructions and using reagents including MG-132 inhibitor.

Three-dimensional spheroids were generated by seeding 1000 INS-1E and BON-1, or 2000 NT-3, cells into each well of a 96-well ULA plate (Corning). For primary cells, 5000 islet cells were seeded into each well of a hanging drop system (#IPS-06-010, Insphero). Three-dimensional cell viability was measured at time 0 (pre-treatment) and at 24 h, 48 h and 72 h post-treatment using the RealTime-Glo™ MT Cell Viability Assay (#G9712, Promega) and following the manufacturer’s instructions. Three-dimensional spheroid size was measured by taking images of the spheroids on the indicated days and analyzing the spheroid size using ImageJ. The combination index CI after the Chou–Talalay method was calculated by using the CompuSyn software [25].

### 2.6. Glucose-Stimulated Insulin Secretion (GSIS)

To assess the capacity of primary mouse islet cells to secrete insulin, a glucose-stimulated insulin secretion (GSIS) assay was performed. The assay was performed in 96-well plates with one 3D microtissue per well. In brief, spheroids were washed three times with medium and then starved for 1 h in 1 mM Glucose. After washing, spheroids were incubated for 60 min with 2.8 mM Glucose (Baseline). Baseline supernatant was collected, spheroids washed and incubated for 60 min with 16.5 mM Glucose (Insulin), and Insulin supernatant was collected. To analyze the amount of secreted insulin, the Baseline and Insulin supernatants were measured using an Ultra Sensitive Insulin ELISA Kit (#90080, CrystalChem-Elk Grove Village, IL, USA) following the manufacturer’s instructions.

### 2.7. RNA Isolation and qPCR

RNA was isolated using the RNeasy Mini Kit (#74104, Qiagen—Hilden, Germany) and RNA concentration was determined by a Spectrophotometer NanoDrop ND-1000 (Thermo Fisher Scientific). cDNA was generated using the High-Capacity RNA-to-cDNA™ Kit (#4387406, Thermo Fisher Scientific). Gene expression was measured using Taqman assays (Thermo Fischer Scientific) according to Supplementary Table S1 and Fast Advanced Master Mix (#4444557, Thermo Fisher Scientific).

### 2.8. Protein Extraction and Western Blotting

Cells were collected and lysed using RIPA buffer (#R0278, Sigma-Aldrich) supplemented with protease (#04693124001, Roche) and phosphatase inhibitors (#04906845001, Roche Diagnostics). Pierce BCA Protein Assay Kit (#23225, Thermo Fisher Scientific) was used to measure protein concentrations. Primary antibodies (Supplementary Table S2) were applied at 4 °C overnight and secondary antibodies (Supplementary Table S2) at room temperature for 1 h. Proteins were visualized using the SuperSignal West Pico Chemiluminescent Substrate Kit (#34080, Thermo Fisher Scientific).

### 2.9. Embedding of Human Tumoroids

For micro-cell-block (MCB) preparation, patient-derived tumoroids corresponding to 3–5 × 10^4^ cells were collected on the day of isolation (D0) and from the 96-well ULA plate at the end of drug screening (D12). Cells were captured in plasma-thrombin clots and fixed, counterstained with Hematoxylin, and embedded in paraffin for sectioning and staining. Embedded material was cut into 2.5-µm-thick serial sections followed by deparaffinization, rehydration and antigen retrieval with the help of an automated immunostainer (Bond RX, Leica Biosystems, Germany). Antigen retrieval was performed in Tris for 30 min at 100 °C for synaptophysin (1:100, 27G12, Novocastra, Leica Biosystem—Deer Park, USA). Primary antibody incubation was 30 min at the specified dilutions. For visualization, a Bond Polymer Refine Detection kit, using DAB (3,3′-Diaminobenzidine), was used as the chromogen. Slides were counterstained with hematoxylin. Scans were acquired with an automated slide scanner Panoramic 250 (3DHistech, Hungary) at 20× magnification. Images were acquired using QuPath software.

## 3. Results

### 3.1. Effect of Buparlisib and Ribociclib on Proliferation and Apoptosis of 2D Cultures of PanNET Cells

With the aim of identifying a novel therapeutic approach for PanNENs, we investigated the effect that the inhibition of two key processes involved in pancreatic tumorigenesis (i.e., the PI3K pathway and cell cycle) would have on the oncogenic features of the tumor cells. Specifically, we tested the PI3K inhibitor buparlisib (BKM120) and the CDK4/6 inhibitor ribociclib (LEE011) alone or in combination in vitro against experimental models representative of well-differentiated and poorly differentiated PanNENs. Specifically, we used the INS-1E cell line (from a rat insulinoma), and the recently established human NT-3 cell line (from a human G2 PanNET) as models of well-differentiated tumors. Indeed, they express markers of NET cells, secrete insulin upon glucose stimulation, and show intermediate (INS-1E) or low (NT-3) proliferation rates [16,21,26]. In our studies, we also included human BON-1 cells, characterized by high proliferation rates, genetic alterations compatible with an aggressive behavior, and partial loss of typical markers of neuroendocrine differentiation [27,28]. Cells were treated with the two drugs alone or in combination, or with DMSO (vehicle control) for 72 h, and then cell proliferation was assessed.

**Figure 1:**
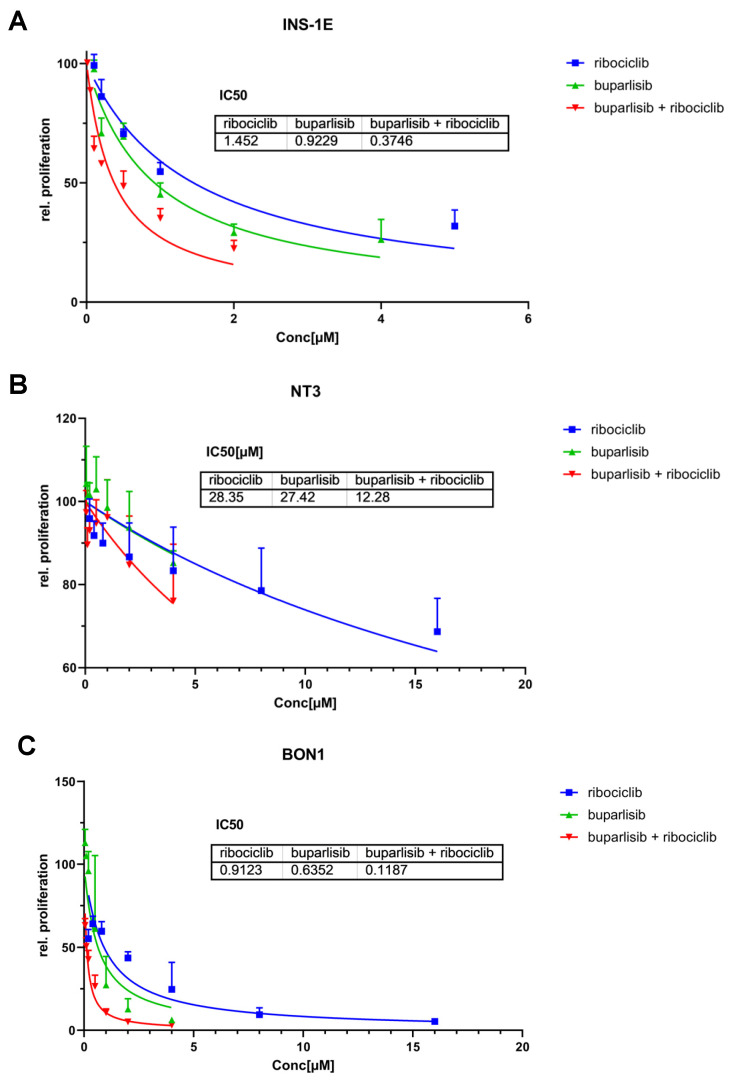
Effect of buparlisib and ribociclib on cell proliferation of INS-1E, NT-3 and BON-1 PanNET cells in 2D culture. INS-1E (**A**), NT-3 (**B**) and BON-1 (**C**) cells were treated with buparlisib, ribociclib, a combination of both drugs or DMSO vehicle control. Cell proliferation was measured after 72 h of treatment. The DMSO control was set to 100% and nonlinear regression was used to determine the IC50. Data shows the mean ± SD from three independent experiments with three technical replicates each.

INS-1E cells responded well to the treatments, and the individual drugs were able to reduce cell proliferation in a dose-dependent manner (Figure 1A). The combination of buparlisib + ribociclib showed the strongest effect, as demonstrated by the lower IC_50_. The single treatment with ribociclib had the weakest effect, and higher doses of this drug were needed to decrease proliferation. The drug combination allowed the reduction of the drug concentration of buparlisib by >70% and of ribociclib by 23% to reach effects that were equally good, or even superior, to the single drugs (Figure 1A). NT-3 cells were in general less sensitive to buparlisib and ribociclib when compared with INS-1E cells (Figure 1B), and the maximum reduction in cell proliferation in all treatment groups was −30%. Comparing the single treatments, buparlisib and ribociclib had a similar effect in NT-3 cells, resulting in comparable IC_50_ values (Figure 1B). Similar to INS-1E cells, the drug combination showed a better effect then the single drug regimens, with an IC_50_ value that was reduced for buparlisib, not for ribociclib.

Both drugs were more effective in BON-1 cells versus the other cell lines, as attested by the lowest IC_50_ values for each agent (Figure 1C). Remarkably, the drug combination led to a strong reduction in proliferation, supporting a synergistic effect of buparlisib and ribociclib in these cells.

**Figure 2:**
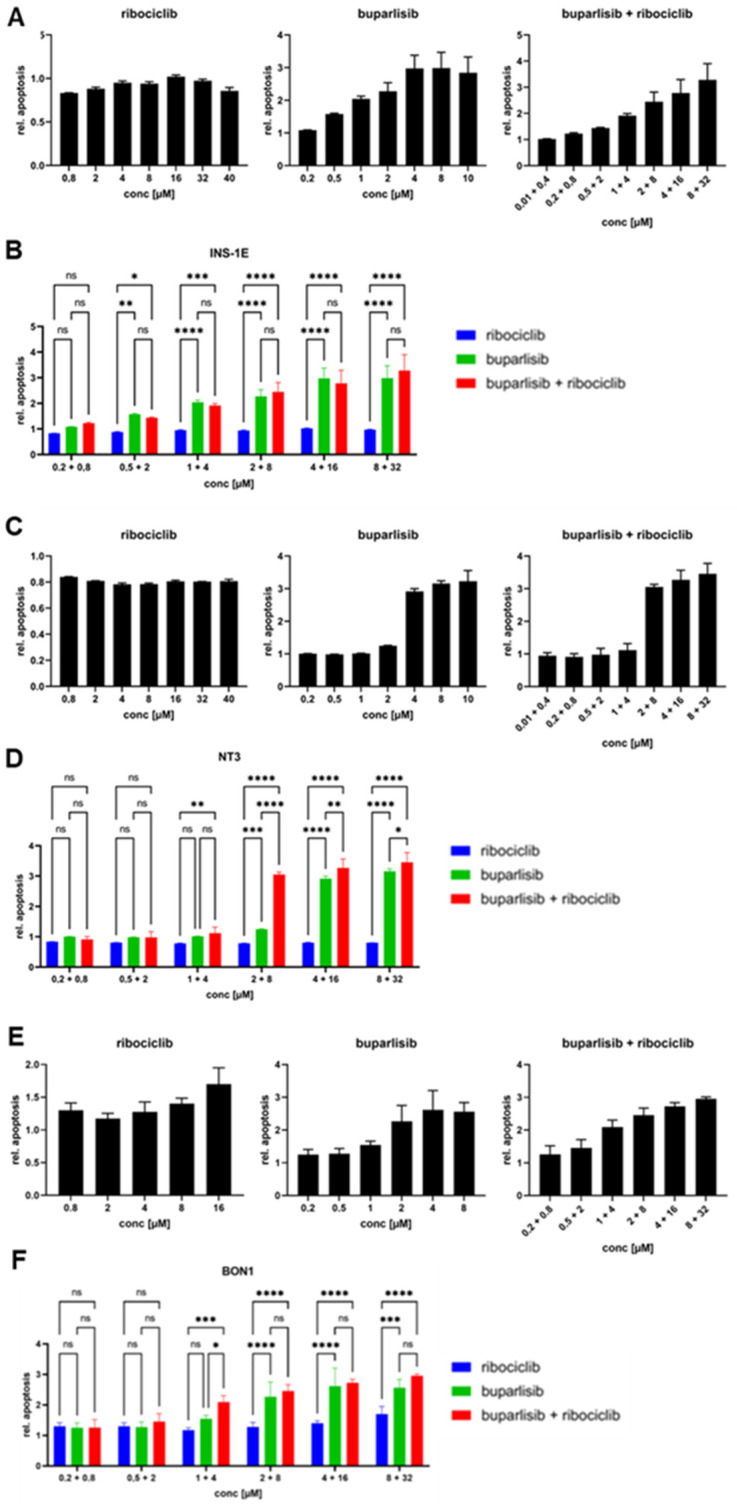
Apoptosis induction upon treatment of INS-1E, NT-3 and BON-1 cells as 2D cultures. INS-1E (**A**,**B**), NT-3 (**C**,**D**) and BON-1 (**E**,**F**) cells were treated with buparlisib, ribociclib, a combination of both drugs or DMSO vehicle control, and caspase 9 activity was measured after 72 h. (**A**,**C**,**E**) A range of concentrations was used to evaluate the effect of the drug treatments. (**B**,**D**,**F**) Comparison of the different treatment regimens. Data was normalized to the DMSO control; the mean ± SD from three independent experiments with three technical replicates each is shown. Two-way ANOVA. ns, not significant; *, *p* < 0.05; **, *p* < 0.01; ***, *p* < 0.001; ****, *p* < 0.0001.

To verify whether the tested drugs not only reduce proliferation but also induce apoptosis of PanNET cells, we measured Caspase9 activity in the three cell lines 72 h after treatment. In INS-1E cells, both buparlisib alone and the drug combination induced apoptosis in a dose-dependent manner, whereas ribociclib alone did not (Figure 2A,B). Direct comparison showed no difference in apoptotic rates between buparlisib as a single agent and the drug combination, indicating that buparlisib is responsible for inducing apoptosis, consistent with its mechanism of action. In NT-3 cells, low concentrations of both buparlisib alone and the drug combination had no pro-apoptotic effects, whereas a clear synergistic effect of both drugs was observed for mid-range concentrations (Figure 2C,D). Ribociclib did not induce apoptosis at any concentration. In contrast, ribociclib at high doses promoted apoptosis in BON-1 cells, while buparlisib alone and the drug combination induced it in a dose-dependent manner (Figure 2E,F).

### 3.2. Effect of Buparlisib and Ribociclib on Downstream Pathway Inhibition in 2D Cultures of PanNET Cells

**Figure 3:**
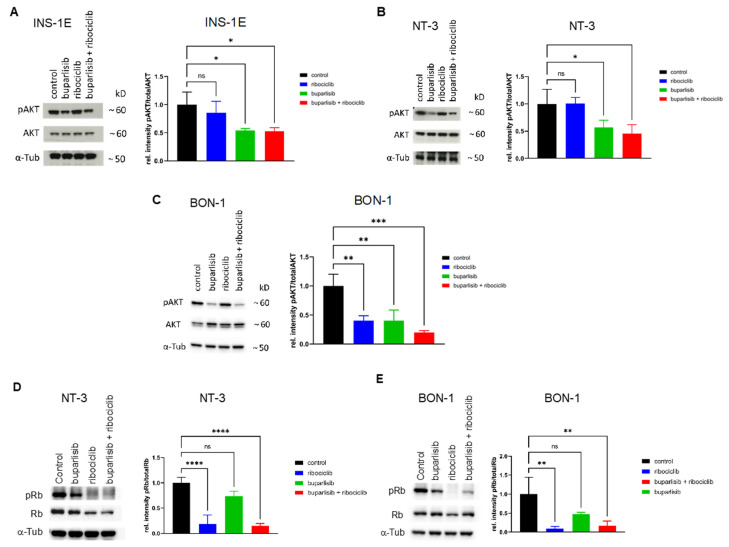
Inhibition of targets of the PI3K pathway and of CDK4/6 confirmed by WB. (**A**–**C**) Expression of phospho-Akt (P-AKT) and total Akt in INS-1E (**A**), NT-3 (**B**) and BON-1 (**C**) cells treated with buparlisib, ribociclib or their combination. α-Tubulin was used as loading control. Shown is one representative immunoblot (out of three). Additionally, the ratio of the band intensities for P-AKT/AKT is given for each cell line. The mean ± SD from three independent experiments is shown. (**D**,**E**) Expression of phospho-Rb (P-Rb) and total Rb in NT-3 (**D**) and BON-1 (**E**) cells treated with buparlisib, ribociclib or their combination. α-Tubulin was used as loading control. One representative immunoblot (out of three) is shown. Additionally, the ratio of the band intensities for P-Rb/Rb is given for each cell line. The mean ± SD from three independent experiments is shown; ns, not significant; *, *p* < 0.05; **, *p* < 0.01; ***, *p* < 0.001; ****, *p* < 0.0001.

We have shown that, in a 2D system, our treatment approach was able to reduce the proliferation and induce apoptosis of both PanNET cell lines. To verify that the observed phenotypes were indeed explained by pathway inhibition and not by unspecific effects, we set out to assess different downstream effectors of the PI3K/AKT or the CDK4/6 pathway. For the former, we analyzed the effect of the drug treatments on the phosphorylation of AKT, a well-known downstream target of the PI3K pathway. Treatment with buparlisib alone and with the drug combination significantly reduced the P-AKT/AKT signal ratio in INS-1E, NT-3 and BON-1 cells (Figure 3A–C). Treatment with DMSO (vehicle control) and ribociclib had no effect on AKT phosphorylation, as expected (Figure 3A–C). This confirmed the downregulation of the PI3K/AKT pathway in PanNEN cells after treatment with the PI3K inhibitor buparlisib. We also performed Western blotting for P-Rb, the target of CDK4/6, which however only gave reliable results for the two human cell lines (NT-3, BON-1). Here, we could see that ribociclib alone or in combination with buparlisib, but not buparlisib alone, decreased the phosphorylation of Rb, as previously reported in other human tumor cell types [29] (Figure 3D,E).

**Figure 4:**
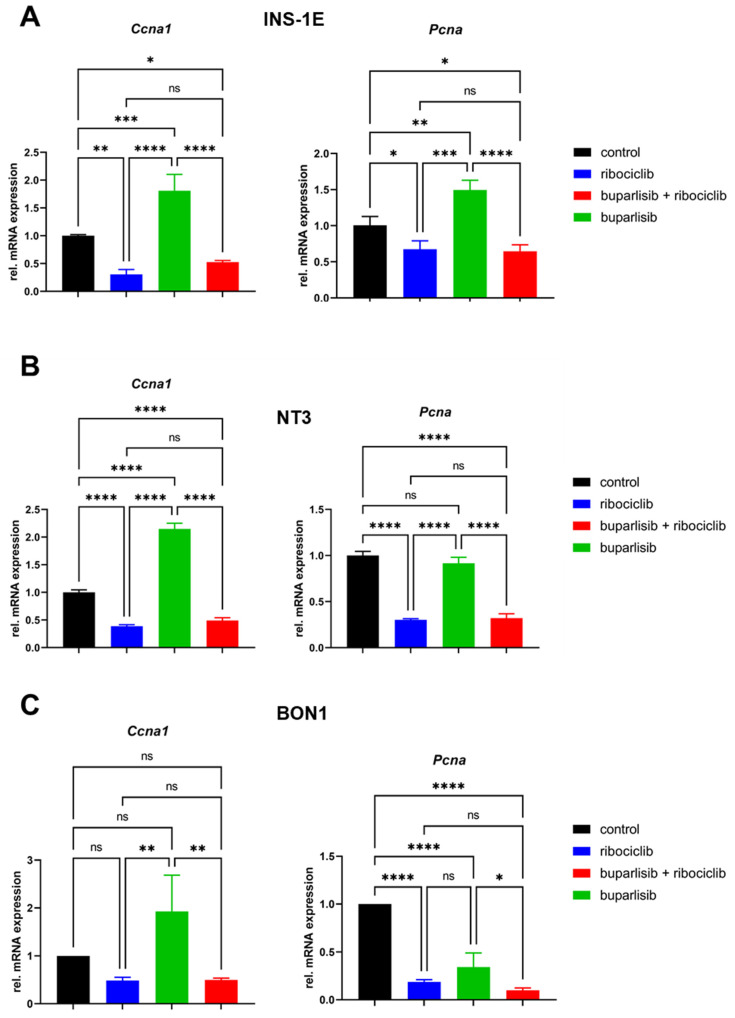
Inhibition of targets of CDK4/6 confirmed by qRT-PCR. Expression of *Ccna1* (cyclin A) and *Pcna* in INS-1E (**A**), NT-3 (**B**) and BON-1 (**C**) cells 72 h after treatment with the indicated drugs. qRT-PCR was carried out using specific TaqMan probes and data were normalized against vehicle control. The mean ± SD of three independent biological replicates is shown. ns, not significant; *, *p* < 0.05; **, *p* < 0.01; ***, *p* < 0.001; ****, *p* < 0.0001 (by 1way ANOVA).

To confirm CDK4/6 inhibition, we analyzed the expression of two genes that are involved in the CDK-P-RB-E2F signaling cascade, namely *Ccna1* (cyclin A1) and *Pcna* (PCNA). The results showed a strong reduction in the expression of these target genes in all lines when treated with ribociclib alone or with the drug combination (Figure 4A–C). In contrast, buparlisib alone only slightly reduced *PCNA* expression in BON-1 cells, while it did not reduce gene expression in the other two cell lines (Figure 4A–C). This data confirmed that the treatment with ribociclib downregulates CDK4/6 signaling in PanNET cells.

### 3.3. Effect of Buparlisib and Ribociclib on the Viability of PanNET Cells Grown as 3D Spheroids

Three-dimensional tumor spheroid cultures (spheroids) have a microenvironment that more closely resembles that of tumors in vivo, and are therefore considered superior to 2D monolayer cultures as in vitro cancer models. Thus, we extended our drug testing to 3D spheroid cultures of the three PanNET cell lines. Using ultra-low-attachment (ULA) plates, INS-1E and BON-1 cells formed round spheres, whereas NT-3-derived spheres were more loose (Supplementary Figure S1). Upon spheroid formation (5 days after plating), cells were treated with buparlisib, ribociclib, their combination or DMSO as vehicle control. Drug concentrations were established by using a 625-fold dilution range to assess the most relevant doses for the 3D spheroids. Cell viability was assessed at different time points after treatment (0 h, 24 h, 48 h and 72 h).

**Figure 5:**
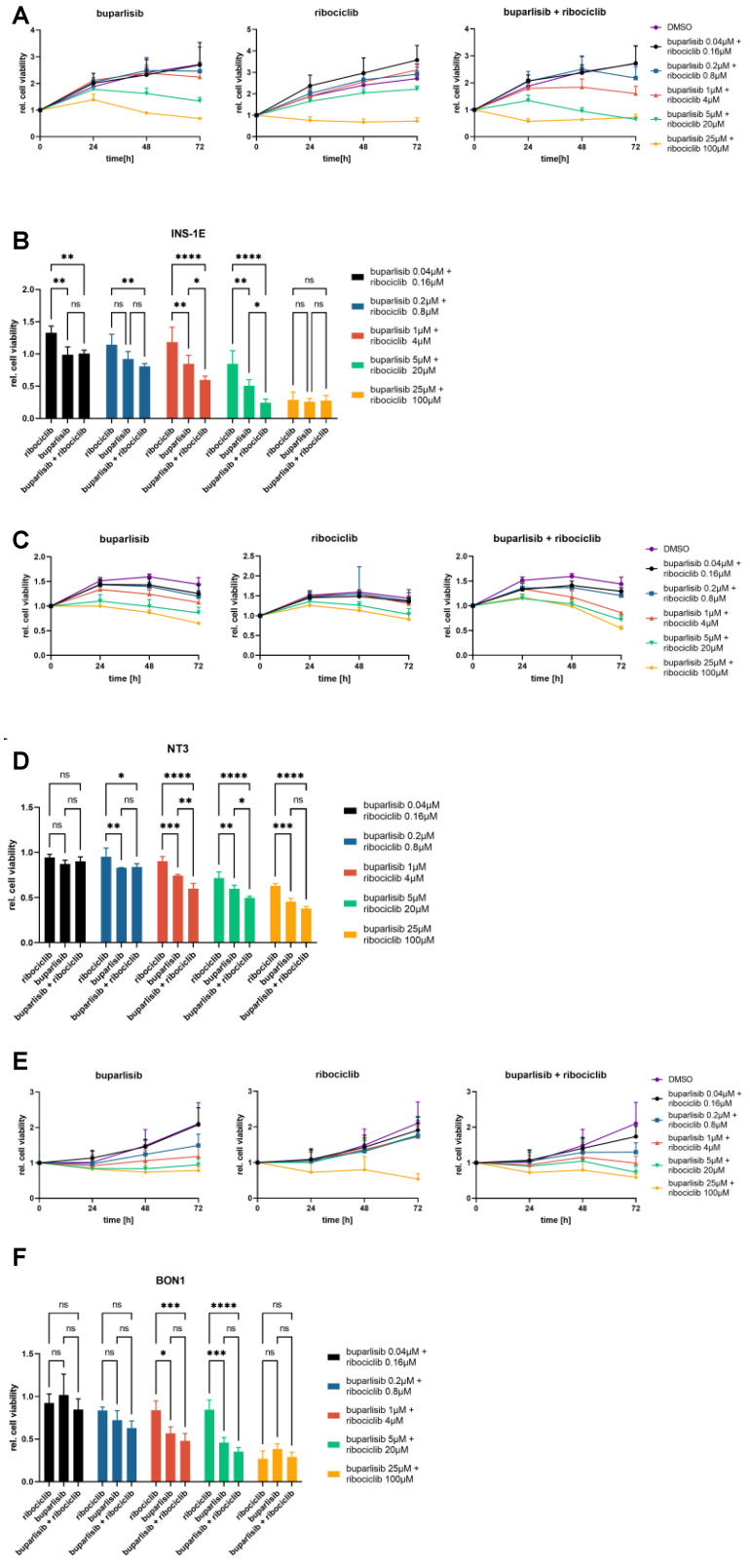
Effect of buparlisib and ribociclib on cell viability of INS-1E, NT-3 and BON-1 PanNET cells in 3D culture. (**A**,**C**,**E**) Cell viability of 3D spheroids of INS-1E (**A**), NT-3 (**C**) and BON-1 (**E**) cells upon treatment with buparlisib, ribociclib, their combination or DMSO vehicle control. A range of concentrations was used to evaluate the effect of the drugs at 24 h, 48 h, 72 h post-treatment. (**B**,**D**,**F**) Relative cell viability of 3D spheroids of INS-1E (**B**), NT-3 (**D**) and BON-1 (**F**) cells at the 72 h timepoint normalized to time 0 and to the DMSO control. Data shows the mean ± SD from three independent experiments with eight technical replicates each. ns, not significant; *, *p* < 0.05; **, *p* < 0.01; ***, *p* < 0.001; ****, *p* < 0.0001 (by 2way ANOVA).

For INS-1E cell spheroids, both drugs were able to reduce cell viability when used individually, with buparlisib showing the strongest effect (Figure 5A,B). At the highest concentrations, both single drugs and their combination reduced cell viability to a minimum. In case of the drug combination, even the second-highest dose (buparlisib 5 µM + ribociclib 20 µM) could strongly reduce cell viability (Figure 5A,B). At the 72 h time point, the drug combination was significantly more effective at inhibiting cell viability in the middle-range doses than each single treatment (Figure 5A,B). Interestingly, the combination therapy showed a superior effect over ribociclib alone even at low doses, as well as a trend towards better efficacy than buparlisib alone.

The overall viability of 3D spheroids of NT-3 cells was lower than that of INS-1E cells, and this resulted in a lower efficacy of the treatments. Nevertheless, both buparlisib and ribociclib, as well as the drug combination, reduced cell viability also of NT-3 cells (Figure 5C,D). At the 72 h time point, the combination worked significantly better than each individual drug at middle-range concentrations, and better than ribociclib at almost all doses (Figure 5C,D).

In BON-1 cells spheroids, there was no additional benefit of the drug combination versus buparlisib alone at 72 h (Figure 5E,F).

To determine whether there was a synergistic effect of the drug combination, we applied the Chou–Talalay method [30,31], where a combination index (CI) is calculated and synergism is defined at CI < 1. We calculated the CI for all three cell lines looking at the EC50 concentration. Interestingly, we found a synergistic effect of the two drugs in all three cell lines with CIs of 0.52 (INS-1E), 0.40 (BON-1) and 0.35 (NT-3).

Altogether, these experiments confirmed the efficacy of the drugs against PanNET cells grown as 3D spheroids.

In addition to assessing cell viability after drug treatment by measuring the reduction of a substrate (=metabolism), we also determined the effects of the drugs on the growth of PanNET cells by following the changes in spheroid size during treatment. Moreover, to assess the long-term effects of the drugs, we longitudinally followed spheroid growth for 14 days after treatment. Spheroid size was measured at day 0 (start of the treatment), and then at days 3, 7, 11 and 14 post-treatment.

**Figure 6:**
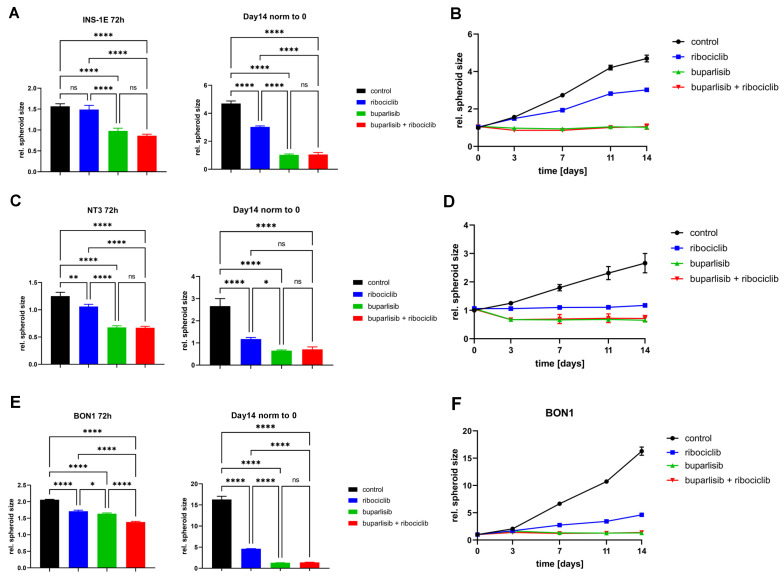
Effect of buparlisib and ribociclib on growth of INS-1E, NT-3 and BON-1 PanNET cells in 3D culture. (**A**,**C**,**E**) Changes in spheroid size at 72 h (3d) and 14d after treatment with buparlisib, ribociclib or their combination in INS-1E (**A**), NT-3 (**C**) and BON-1 (**E**) cells. (**B**,**D**,**F**) Effect of the treatments on the relative spheroid size (versus day 0) over the course of 14 days in INS-1E (**B**), NT-3 (**D**) and BON-1 (**F**) cells. Data shows the mean ± SD from three independent experiments with eight technical replicates each. ns, not significant; *, *p* < 0.05; **, *p* < 0.01; ****, *p* < 0.0001 (by 1way ANOVA).

INS-1E spheroids showed a progressive darkening of the center of the sphere following the combination treatment (Supplementary Figure S1). At the 72 h timepoint, both buparlisib and the drug combination suppressed growth, while ribociclib did not (Figure 6A,B). An effect on the size and on the opacity of the INS-1E spheroids (versus control) could be appreciated already 72 h after treatment with the drug combination (Figure 6A,B and Supplementary Figure S1). After 14 days, the anti-proliferative effect of both buparlisib and the drug combination was more noticeable: these two regimens completely stopped the growth of INS-1E spheroids (Figure 6A,B). At the 14 days time point, ribociclib had significantly inhibited spheroid growth versus vehicle control (Figure 6B).

Similar to INS-1E cells NT-3 cell spheroids also showed a clear reduction in size and a progressive darkening of the center during treatment (Figure 6C,D and Supplementary Figure S1). After 72 h, buparlisib, ribociclib and their combination suppressed NT-3 spheroid growth, with buparlisib and the combination showing the strongest inhibition (Figure 6C,D). While ribociclib stopped cell growth versus day 0, buparlisib and the combination treatment even led to a slight reduction of spheroid size (Figure 6C). Ribociclib as a single agent was more effective against NT-3 cell spheroids than INS-1E spheroids already after 72 h and its anti-tumor effect was even more pronounced after 14 days (Figure 6C,D). At the this time point, no increase in size was measured upon buparlisib and combination treatment (Figure 6C,D).

Similarly, a strong effect of buparlisib and the drug combination was observed in BON-1 cells, and was especially prominent at the 14-day time point (Figure 6E,F). Ribociclib as a single agent was able to suppress the growth of BON-1 spheroids, especially considering the high proliferation rate of these cells when vehicle-treated (Figure 6E). Thus, in the long term, buparlisib and the combination buparlisib + ribociclib completely suppressed spheroid growth in all PanNET cell lines, whereas ribociclib alone reduced cell growth, with NT-3 and BON-1 being especially affected.

### 3.4. Effect of Buparlisib and Ribociclib on Viability, Growth and Function of Islet Microtissues Derived from Mice with Men1 Gene Deletion

**Figure 7:**
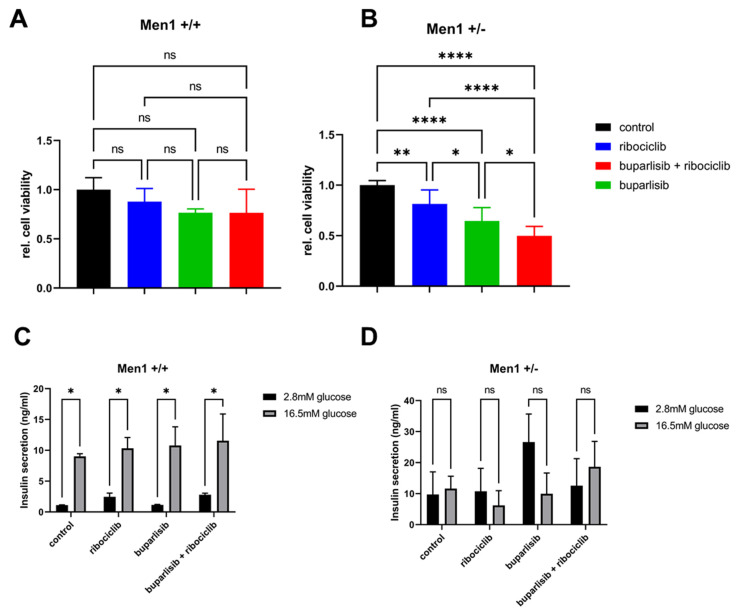
Effect of buparlisib and ribociclib on growth and glucose-stimulated insulin secretion of primary 3D islet microtissues from *Men1* knockout and control mice. (**A**,**B**) Primary islets were isolated from age-matched (18 months) heterozygous *Men1^T/+^* (**A**) and *Men1^+/+^* control (**B**) mice. Single cells were obtained from the islets and 3D microtissues generated using a hanging-drop system. Spheroids were treated with DMSO, or buparlisib and ribociclib alone or in combination, for 72 h after spheroid formation and cell viability was measured. The relative cell viability normalized to the initial measurement and the DMSO control is shown. Data shows the mean ± SD from primary cells of nine *Men1^T/+^* mice (with tumors) and five *Men1^+/+^* mice with 4–14 technical replicates each (depending on total amount of cells). One-way ANOVA. Ns, not significant; *, *p* < 0.05; **, *p* < 0.01; ****, *p* < 0.0001. (**C**,**D**) Glucose-stimulated insulin secretion of primary 3D microtissues. Primary islets were isolated, processed and treated for 72 h as above. They were serum-deprived, incubated with low glucose (2.8 mM, baseline level) or with high glucose (16.5 mM) for 1 h. Then, insulin secretion was assessed using a specific ELISA assay using the supernatants. Data shows the mean ± SD from primary cells of three *Men1^T/+^* and 3 *Men1^+/+^* mice with three technical replicates each. One-way ANOVA. ns, not significant; *, *p* < 0.05.

Buparlisib and ribociclib were found to significantly suppress the proliferation/viability of both 2D and 3D cultures of established PanNET cell lines. We then wondered whether these drugs could also be effective against primary PanNET cells. Mice heterozygous for the deletion of exon 3 of the *Men1* gene exon 3 (*Men1^T/+^)*, develop insulinomas (β cell tumors) from the age of 12 months, which closely resemble their cognate human tumors [23]. Thus, we employed these mice to test the efficacy of our drugs. We isolated islets from *Men1^T/+^* mice (*n* = 9) at the age of 18 months. To verify whether the drugs also have an effect on healthy islets, we also isolated and treated islets from control *Men1*^+/+^ (*n* = 5) mice. To overcome the issue that freshly isolated islets differ in size and could affect therapy response, islets were digested and then reconstituted as 3D microtissues (pseudo-islets) containing the same number of cells (Supplementary Figure S2). Pseudo-islets of both groups were treated with 5 µM buparlisib, 20 µM ribociclib or their combination (buparlisib 5 µM + ribociclib 20 µM). Interestingly, neither the single drugs nor their combination significantly affected the viability of healthy islet cells of *Men1*^+/+^ littermates (Figure 7A). In contrast, treatment with buparlisib or ribociclib was effective at reducing the viability of PanNET cells from *Men1^T/+^* mice (Figure 7A), with the former drug working significantly better than the latter. The drug combination was the most effective treatment (Figure 7A). The difference in sample size between the two mouse groups might affect the statistics.

Glucose-stimulated insulin secretion is one of the most important physiological features of the pancreatic islets, which is carried out by the pancreatic β cells, the cells of origin of the tumors developing in *Men1^T/+^* mice. In view of a possible translation of these drugs in clinical practice, it is important to know whether they perturb insulin secretion. Thus, we generated pseudo-islets from islets isolated from *Men1^T/+^* and *Men1*^+/+^ mice, and treated them with buparlisib and ribociclib, alone or in combination, as done for the cell viability assays. After 72 h of treatment, spheroids were starved before measuring baseline insulin secretion (incubation with 2.8 mM glucose), or secretion upon stimulation with high glucose (incubation with 16.5 mM glucose). As expected, the low baseline insulin secretion of pseudo-islets from *Men1*^+/+^ significantly increased upon glucose stimulation (Figure 7B). The treatment with both drugs, alone or in combination, did not affect insulin secretion in control *Men1*^+/+^-derived pseudo-islets (Figure 7B). *Men1^T/+^* pseudo-islets showed a higher baseline insulin secretion compared to the pseudo-islets of *Men1*^+/+^ mice (Figure 7B). With the exception of an increase in basal insulin levels in pseudo-islets from *Men1^T/+^* mice, we could not see an effect of the glucose stimulation in any other condition (Figure 7B). These results suggest that insulin secretion is perturbed in pseudo-islets of tumor-bearing heterozygous *Men1^T/+^* mice. As the drugs showed no effect on the ability of pseudo-islets of *Men1*^+/+^ mice to secrete insulin upon glucose stimulation, it can be concluded that these treatments do not interfere with this physiological function of pancreatic islets.

### 3.5. Effect of Buparlisib and Ribociclib on the Viability of Human-Derived PanNET 3D Tumoroids

**Figure 8:**
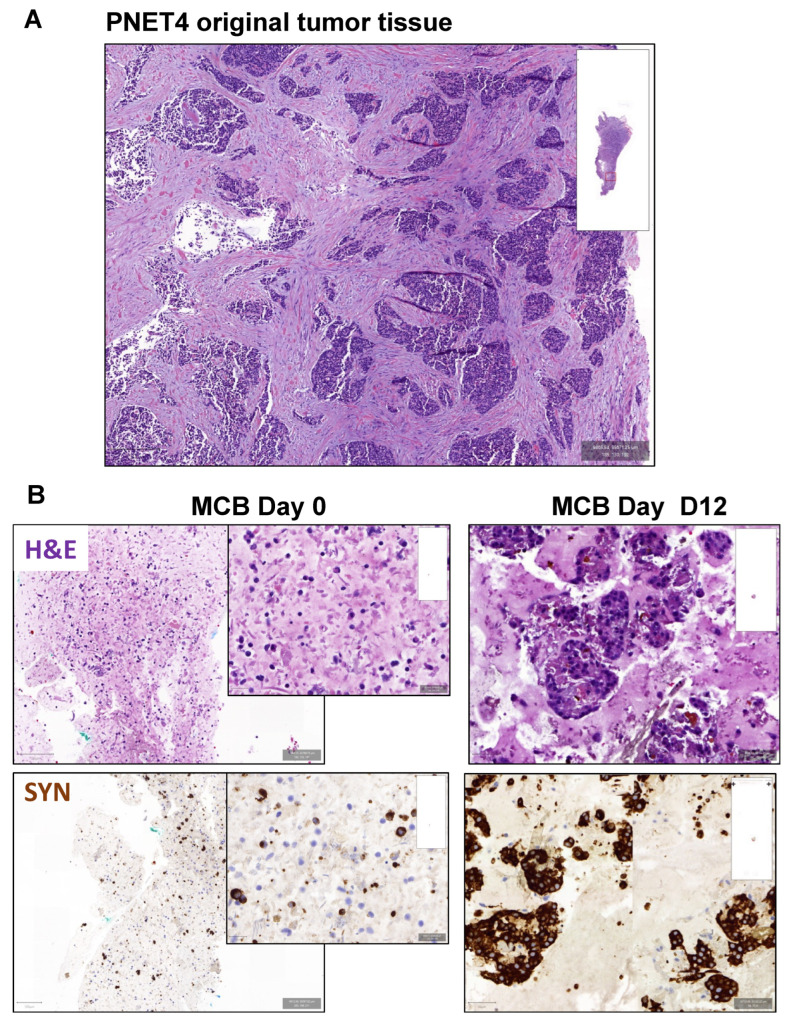

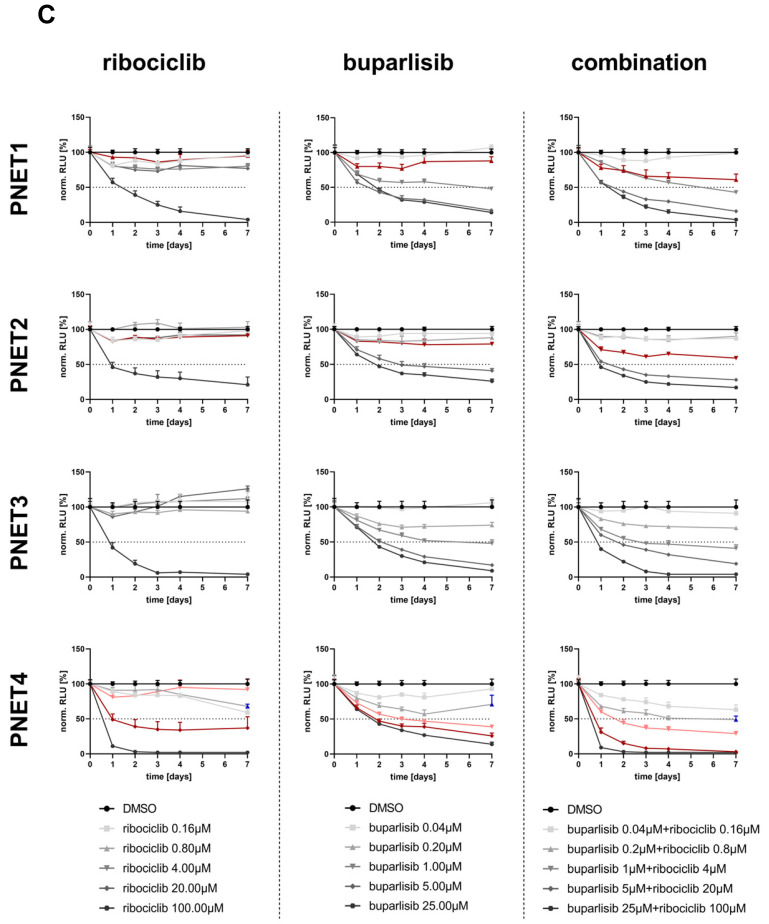
Effect of buparlisib and ribociclib on cell viability of primary human 3D tumoroids. (**A**,**B**) Representative images of sample PNET4. (**A**) H&E staining (H&E) of the original tumor tissue. Scale bar (250 μm). (**B**) H&E staining and staining for synaptophysin of micro-cell-block samples from the day of isolation (Day 0) and DMSO-treated samples 12 days post-isolation (Day 12). MCB = Micro-cell-block; SYN = Synaptophysin. Scale bar MCB Day 0 (100 μm), scale bar inset (20 μm), scale bar MCB Day 12 (50 μm). (**C**) Cell viability curves of human tumoroids PNET1, PNET2, PNET3 and PNET4 treated with different concentrations of buparlisib, ribociclib and their combination for 7 days. For clarity, each single treatment and the combination are shown separately. Drug concentrations are reported below the graphs. Drug concentrations for which the combination was more effective than the individual drugs are illustrated in red. Data were first normalized per-well using a RTG baseline measurement for each individual well and then normalized to the average of the corresponding DMSO control of the respective day. Data represent means ± SEM (*n* = 1 per patient, three technical replicates). RLU, relative luminescence unit.

To determine whether the antitumor effect of buparlisib and ribociclib harbors translational relevance, we treated patient-derived 3D tumoroids obtained from four tumors of four patients (two primary tumors and two liver metastases) with the two drugs alone or in combination. Patient information is available in Supplementary Table S3. Tumor tissues were digested and reconstituted as 3D microtissues (tumoroids), which were then treated with various drug concentrations over a 7-day period (drug doses are reported in Figure 8C). Part of these microtissues were embedded on the day of isolation (D0) and 12 days later (D12) to verify morphology and marker expression. As previously described [22], patient-derived tumoroids in vitro retained both the histomorphology in the original tumors from which they were derived, as well as the expression of neuroendocrine cell markers (e.g., synaptophysin) (Figure 8A,B).

Comparable to our findings using established PanNET cell lines and primary islet microtissues from *Men1^T/+^* mice, we observed a dose-dependent decrease in viability when treating the human 3D tumoroids with the two drugs (Figure 8C, Supplementary Figure S3). Buparlisib as a single agent was more effective than ribociclib in all samples except PNET4. Seven days after treatment, the combination of buparlisib with ribociclib was more effective than the individual treatments in samples PNET1 and PNET2, and showed a synergistic effect in PNET4, as calculated using the Chou–Talalay method [30,31] (Figure 8B, Supplementary Figure S3). The combination showed a superior antitumor effect in three out of four samples for certain drug concentrations, which are indicated in red in the viability curves (Figure 8C), and marked with an asterisk in Supplementary Figure S3. Sample PNET4 displayed the strongest sensitivity to the drug combination, mostly due to a stronger response to ribociclib versus the other primary cultures. Interestingly, in PNET4 the lower doses of buparlisib lost their efficacy with time, thereby showing an increased cell viability at the 7-day time point compared with earlier time points (Figure 8C). In contrast, the same doses of buparlisib, when combined with ribociclib, did not led to an increase in cell viability, but actually to a further decrease when compared to earlier time points (Figure 8C).

## 4. Discussion

In our study, we targeted PI3K and CDK4/6 signaling and found that this approach holds promise for the treatment of PanNETs. Indeed, the combination of buparlisib and ribociclib leads to additive/synergistic antitumor effects in established PanNET cells (in both 2D and 3D culture systems), as well as in primary islet tumor microtissues from *Men1^T/+^* knockout mice. Importantly, this drug combination also exhibited antiproliferative effects in patient-derived primary PanNET 3D tumoroid cultures.

For our studies, we selected cell lines representative of well-differentiated (INS-1E, NT-3 cells) and poorly differentiated (BON-1) PanNENs, and employed clinically relevant doses of both drugs in view of a potential translation of our findings [32,33]. Overall, the combination of buparlisib with ribociclib was more effective than each agent alone at suppressing the proliferation of all cell lines in 2D, with the effect being synergistic for BON-1 cells (proliferation) and for NT-3 cells (apoptosis) and additive in the remaining settings. Buparlisib as a single agent was more effective than ribociclib at inhibiting cell proliferation, with the exception of NT-3 cells, which were equally sensitive to both drugs. The data on the efficacy of buparlisib against BON-1 cells in vitro are in agreement with a previous study [18]. The higher responsiveness of NT-3 cells to CDK4/6 inhibition fits with the data obtained in *Men1*^+/−^; *Cdk4*^−/−^ double knockout mice, where PanNETs formation dependent on *Men1* gene inactivation was abrogated [15]. Indeed, NT-3 cells have a non-functional menin (the product of the *MEN1* gene) [21], hence are “addicted” to enhanced cell division. Thus, blocking CDK4 activity (i.e., inhibiting cell cycle progression) in these cells is predicted to have a strong negative effect on cell proliferation.

The ability to promote tumor cell death is a highly desirable feature of anti-cancer therapies and has clinical relevance. Thus, we assessed the ability of both drugs to induce apoptosis. In both INS-1E and BON-1 cells, apoptosis was exclusively mediated by buparlisib. In contrast, a synergistic effect of buparlisib and ribociclib to promote apoptosis in NT-3 cells was observed, in line with the higher sensitivity of these cells to CDK4/6 inhibition.

The response of PanNET cells to the selected drugs correlated with the inhibition of the corresponding downstream targets: P-AKT for buparlisib, and P-Rb for ribociclib. Ribociclib also elicited the downregulation of *Ccna1* and *Pcna,* direct targets of E2F transcription factors, which become active following Rb phosphorylation. Therefore, the effects of the drugs on proliferation and apoptosis are mediated by downstream pathway inhibition and not by off-target effects.

Three-dimensional spheroids, which better mimic the physiological microenvironment of solid tumors, were shown to have a higher predictive value of therapy efficacy than standard 2D cultures [34,35,36], and are currently the preferred in vitro cancer model for drug testing. For this reason, we extended our therapy studies to 3D spheroid cultures of PanNET cells. Using a 14-day treatment regimen, we observed a synergistic effect of the combination buparlisib and ribociclib on the suppression of the viability of all three cell lines, and on the reduction of spheroid size of INS-1E and NT-3 cells, but not BON-1 cells. The drug concentrations that elicited the strongest effect were higher than in the 2D cell culture setting, a fact that has been previously reported and results from the structure of the spheroids [34,37]. Similar to the 2D culture system, NT-3 were also more responsive to ribociclib when grown as 3D organotypic cultures. Currently, we have no explanation as to why ribociclib is less effective against 3D versus 2D cultures of BON-1 cells.

To further validate the efficacy of our treatment approach, we extended our analyses to primary PanNET cells from *Men1^T/+^* mice grown as 3D islet microtissues. The drugs reduced the viability of primary PanNET microtissues both as single agents and in combination, with the latter being the most effective condition. Interestingly, no significant effect of the drugs (neither alone nor in combination) was observed on the viability of primary 3D microtissues established from islets of wild-type littermates (*Men1^+/+^*), thereby pointing to a selective efficacy of the treatment regimen for the tumor cells versus wild-type islets. Additionally, treatment of wild-type islet microtissues with the drugs did not significantly affect insulin secretion, the most important physiological role of pancreatic β cells, which are the major cellular components of the islets and the cells of origin of tumors in this mouse model. Three-dimensional PanNET microtissues from *Men1^T/+^* mice failed to respond to glucose, suggesting β cell dysfunction.

In addition to primary islet cells from mice, we set out to test out treatment approach in patient-derived primary 3D tumoroid cultures. Specifically, we established 3D tumoroids from four human PanNET tissues (two primary tumors and two metastases), and treated them with buparlisib and ribociclib as single agents or in combination. Importantly, we confirmed that the patient-derived tumoroids preserved the histomorphology and the expression of neuroendocrine cell markers of the tumors from which they were derived. Similar to the results obtained using PanNET cell lines and murine primary islet cells, our drugs could also reduce the viability of human primary PanNET cells, with the combination of buparlisib and ribociclib giving the strongest results in three out of four patients’ samples. This effect was most remarkable in PNET4, where we saw a strong synergistic effect of our combination treatment 7 days after treatment. Overall, the different patient-derived primary PanNEN cultures showed a variable sensitivity to our drugs, which is not entirely unexpected given the documented heterogeneity of these tumors.

The PNET4 sample was obtained from the liver metastasis of an aggressive and recurrent PanNET developing in a female patient (37 years) which did not respond to standard sunitinib treatment. Remarkably, PNET4 showed the highest sensitivity to ribociclib when compared to the other human primary PanNENs. No information about the genetic makeup of the tumors from which the primary cultures were derived is available. This, combined with the low number of samples analyzed, limits the possibility of correlating the drug response to specific genetic mutations/molecular subtypes in the primary PanNETs. Nevertheless, our analysis demonstrates that this therapeutic approach was effective in three out of four samples, eliciting the same effect as higher doses of buparlisib alone. The possibility to decrease buparlisib concentrations is interesting for future clinical applications as it would reduce the drug’s side effects. Moreover, the combination buparlisib and ribociclib might be an option for tumors that showed resistance to standard therapies. The analysis of additional primary human PanNETs is required to confirm our findings.

The activation of the PI3K/Akt/mTOR pathway in PanNET tumorigenesis led to the clinical use of drugs targeting this pathway. The drug for which more information is currently available is everolimus, an inhibitor of the mTORC1 complex [38], which has been FDA-approved for the treatment of patients with unresectable, locally advanced or metastatic PanNETs [39]. Indeed, everolimus significantly prolonged progression-free survival (PFS) in a large Phase III trial of advanced PanNETs [40]. However, inhibition of mTORC1 removes negative feedback in Akt, thereby causing undesired Akt activation and leading to therapy resistance [41]. Therefore, agents blocking the pathway upstream at the level of PI3K are expected to bypass therapy resistance.

## 5. Conclusions

By demonstrating that the combination treatment of buparlisib with ribociclib is effective against PanNET cell lines representative of tumors with different differentiation states, and having different molecular signatures, as well as against primary mouse and human PanNEN cells, our studies provide the rationale for the clinical implementation of drugs co-targeting PI3K and CDK4/6 signaling pathways in PanNETs.

## Data Availability

All data that support the findings of this study are available from the corresponding authors upon reasonable request.

## Supplementary Materials

The following supporting information can be downloaded at: https://www.mdpi.com/article/10.3390/cancers14225481/s1, Figure S1: Effect of the combination buparlisib and ribociclib on 3D spheroid cultures of the PanNET cell lines; Figure S2: Primary 3D microtissues from mouse pancreatic islets (pseudo-islets). Figure S3: Representative primary human 3D tumoroids; Figure S4: Effect of buparlisib, ribociclib and their combination on human-derived PanNET 3D tumoroids; Table S1: List of TaqMan gene expression assays used for qPCR; Table S2: List of antibodies used for Western blotting; Table S3: Clinico-pathological features of the patients from whom PanNEN tissues were obtained at surgery to establish primary cultures.
