# Supplementary material for: Combined Targeting of Pathogenetic Mechanisms in Pancreatic Neuroendocrine Tumors Elicits Synergistic Antitumor Effects
Source: Cancers (Basel). 2022 Nov 8;14(22):5481. doi: 10.3390/cancers14225481 (PMC9688197; doi:10.3390/cancers14225481)
Supplement: Supplementary file 1 [file cancers-14-05481-s001.zip › cancers-1995750-supplementary.pdf]

Supplementary Tables

Supplementary Table S1: List of TaqMan gene expression assays used for qPCR

Assay ID	Gene name	Dye	Species
Hs00187842_m1	B2M	FAM-MGB	human
Hs00171105_m1	ccna1	FAM-MGB	human
Hs00427214_g1	pcna	FAM-MGB	human
Rn00560865_m1	B2M	FAM-MGB	rat
Rn01761348_m1	ccna1	FAM-MGB	rat
Rn01514538_g1	pcna	FAM-MGB	rat

Supplementary Table S2: List of antibodies used for Western blotting

Type	Antibody	Clonality	Company	ID	Dilution
Primary	Akt Antibody	Rabbit	Cell Signaling Technology	9272	1/1000
Primary	Phospho-Akt (Ser473) (D9E) XP®	Rabbit	Cell Signaling Technology	4060	1/2000
Primary	Rb (4H1) Mouse mAb	Mouse	Cell Signaling Technology	9309	1/2000
Primary	Phospho-Rb (Ser807/811) Antibody	Rabbit	Cell Signaling Technology	9308	1/1000
Primary HRP	α-Tubulin (DM1A) Mouse mAb (HRP Conjugate)	Mouse	Cell Signaling Technology	12351	1/5000
Primary insulin	Insulin polyclonal	Guinea pig	Dako	A0564	1/750
Primary glucagon	Glucagon polyclonal	Rabbit	Dako	A0565	1/1500
Secondary	Rabbit IgG HRP Linked Whole Ab		GE Healthcare	NA934 V	1/2000
Secondary	F(ab')2-Goat anti-Mouse IgG (H+L) Highly Cross-Adsorbed Secondary Antibody, HRP		Invitrogen	A24518	1/2000
Secondary IF*	Rabbit IgG [H+L] fluorescein isothiocyanate–conjugated		Invitrogen	F-2765	1/200
Secondary IF*	Guinea pig IgG [H+L] Alexa Fluor 555–conjugated		Invitrogen	A-21453	1/200

*immunofluorescence

Supplementary Table S3: Clinico-pathological features of the patients from whom PanNEN tissues were obtained at surgery to establish primary cultures.

Patient	Sex	Age	Location	Ki-67	T	N	M
PNET1	Female	55	Pancreas primary	2.5%	T2	N0	M0
PNET2	Female	29	Pancreas primary	4.0%	T2	N0	M0
PNET3	Male	65	Liver metastasis	15%	Tx	Nx	M1
PNET4	Female	37	Liver metastasis	10%	Tx	Nx	M1

Supplementary Figures

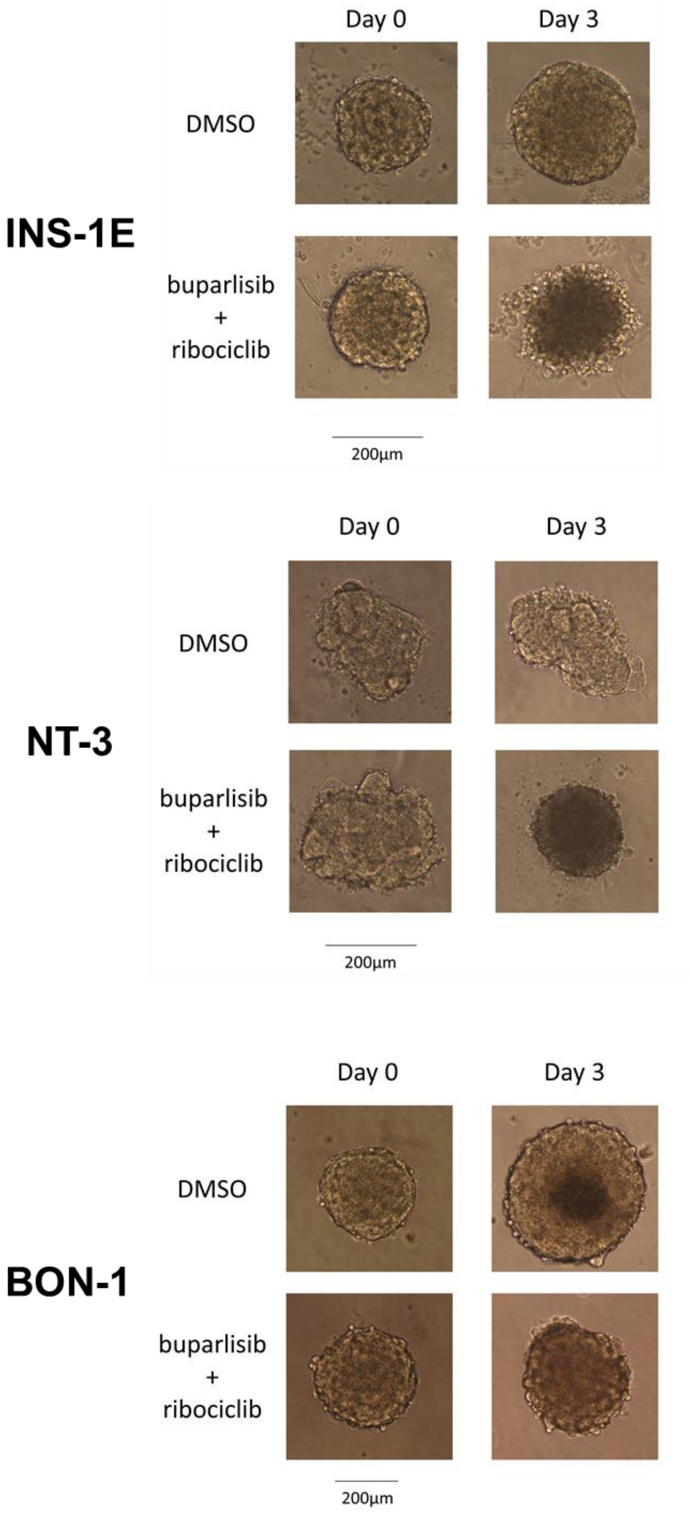

Supplementary Figure S1. Effect of the combination buparlisib and ribociclib on 3D spheroid cultures of the PanNET cell lines. INS-1, NT-3 and BON-1 cells were plated in ultra-low attachment plates and 3-5 days later (Day 0) they were treated with DMSO vehicle or with the combination buparlisib and ribociclib for 3 days. Pictures of representative spheroids were taken at Day 0 and Day 3 under the light microscope maintaining the same magnification.

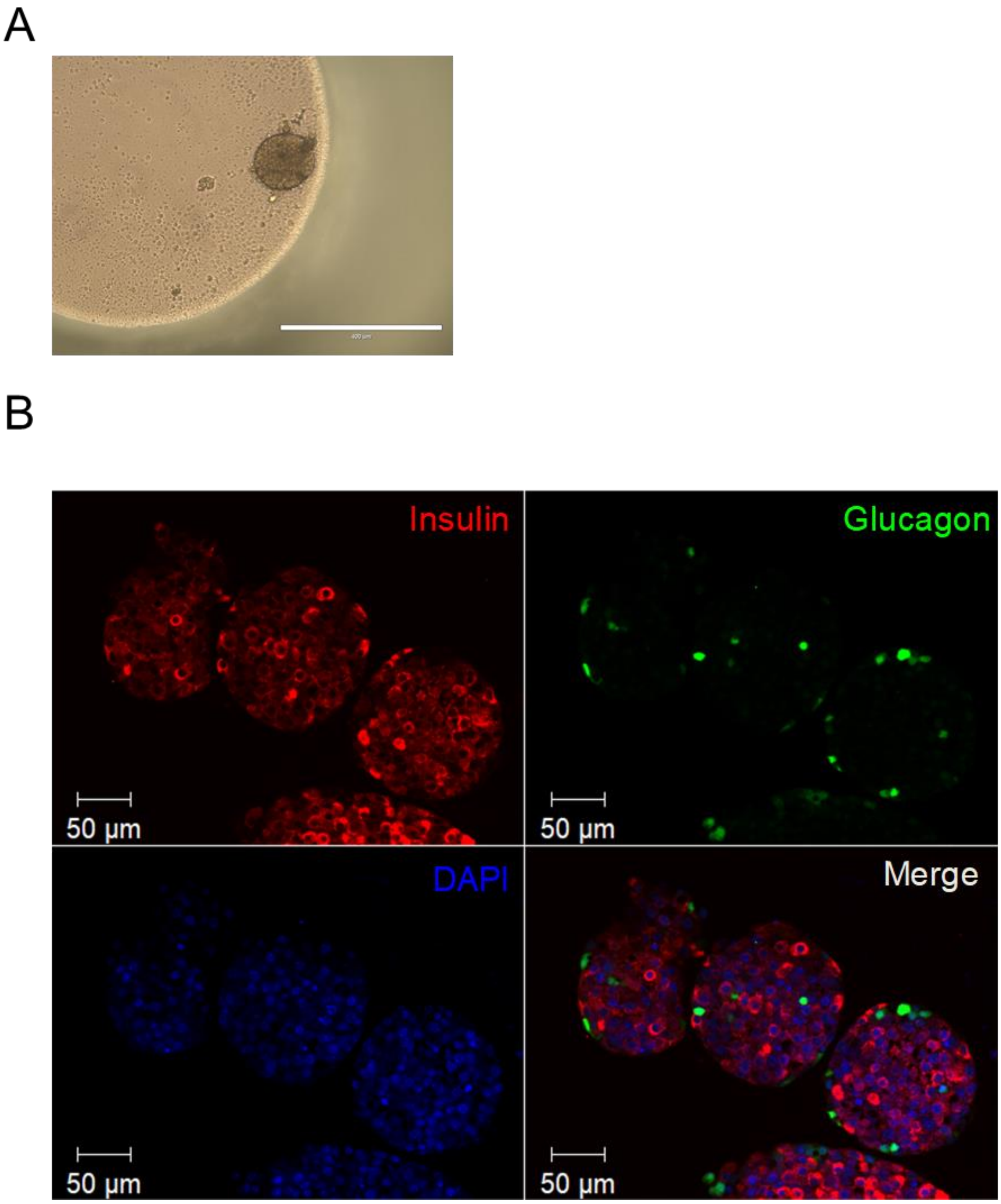

Supplementary Figure S2. Primary 3D microtissues from mouse pancreatic islets (pseudo-islets). (A) Mouse islets were islets were digested and then reconstituted as 3D microtissues (pseudo-islets) using the hanging drop system (Gravity plates). Each well contained the same number of cells. **(B)** Using this culture system, the pseudoislets re-organize themselves as isolated primary islets. Pictures of representative pseudoislets from *Men1*$^{+/+}$ mice were taken at Day 5 under the light microscope (A) or after immunofluorescent stainings with the islet cell markers insulin and glucagon (B). Size bar: 400μm (A); 50μm (B).

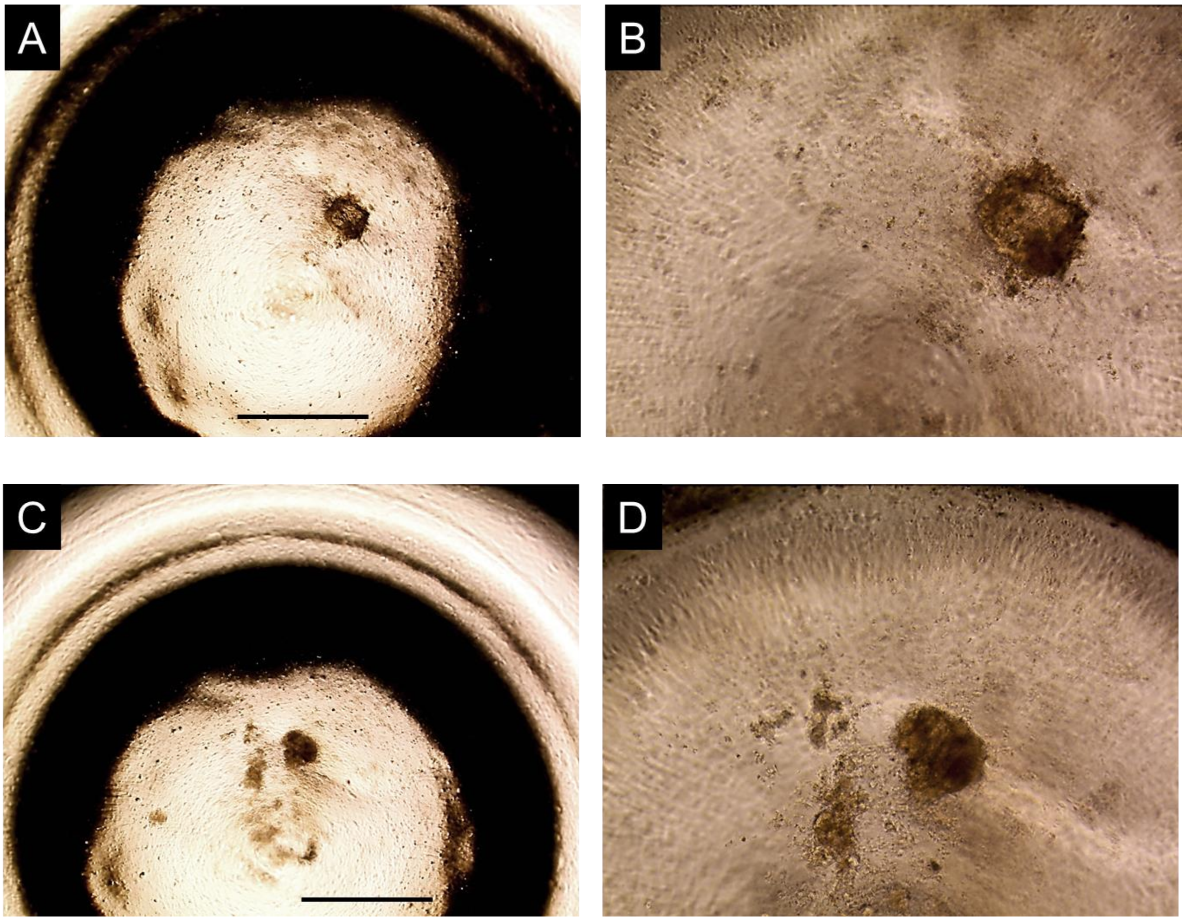

Supplementary Figure S3. Representative primary human 3D tumoroids. (A,B) Images of the PNET1 sample. Human tumor tissues were dissociated as reported in the Materials & Methods and the same number of cells was plated in ultra-low attachment plates. Brightfield pictures were taken using the EVOS system 7 day after treatment with DMSO vehicle (controls). **(C,D)** Images of the PNET2 sample. (A, C) Size bar: 400μm. Original magnification: 4X (A,C); 10X (B,D).

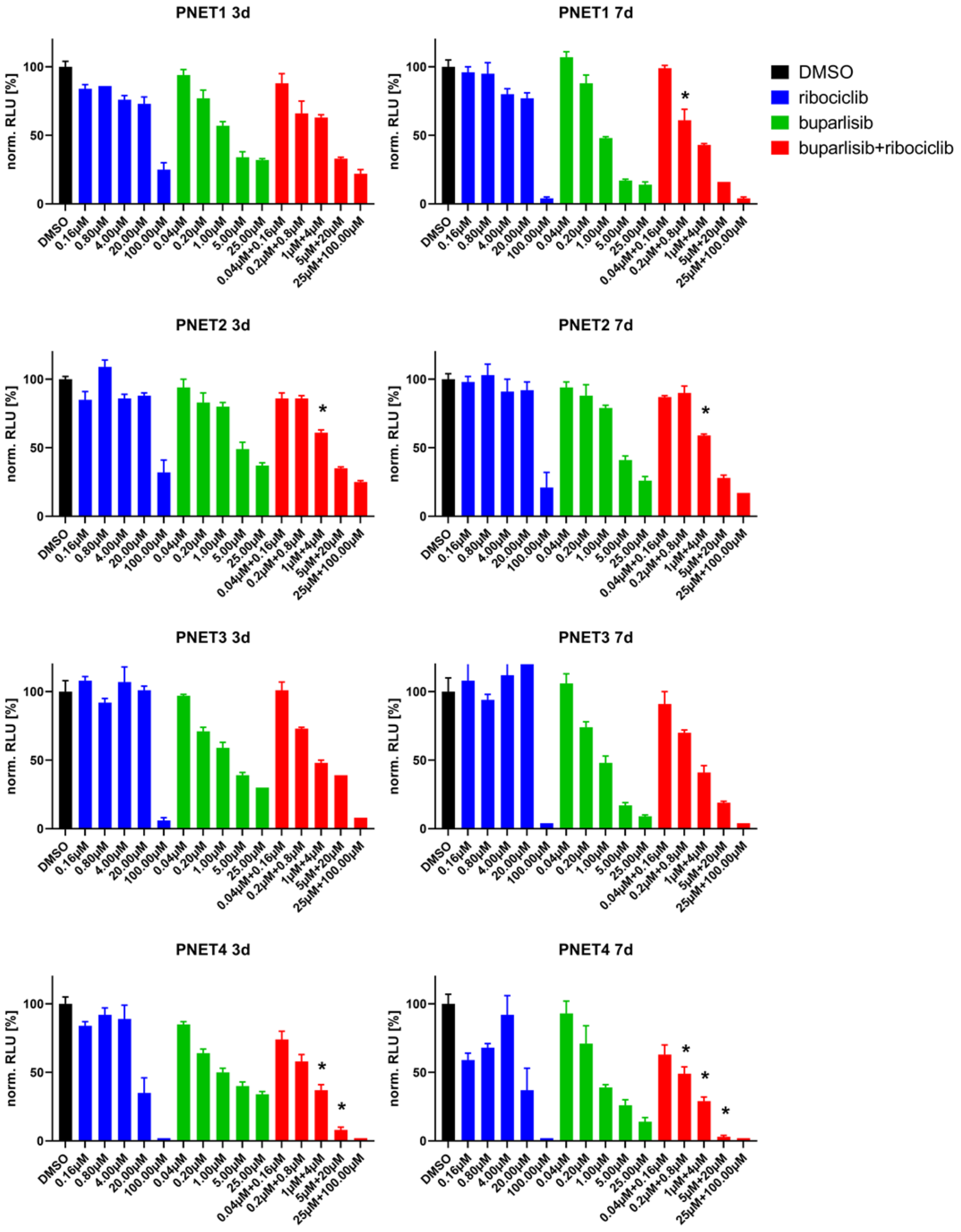

Supplementary Figure S4. Effect of buparlisib, ribociclib and their combination on human-derived PanNET 3D tumoroids. Cell viability of human tumoroids PNET1, PNET2, PNET3 and PNET4. Primary tumor cells were plated and after 5 days they were treated with the indicated concentrations of buparlisib or ribociclib alone or in combination for a total of 7 days. Here are shown the values for the 3-day time point (3d) and the 7-day (7d) time point. Data were first normalized per-well using a RTG baseline measurement for each individual well and then normalized to the average of the corresponding DMSO control of the respective day. Data represent means ± SEM (n = 1 per patient, three technical replicates). RLU, relative luminescence unit. *, significant decrease in cell viability *versus* single treatments, $P<0.05$.
